# Pakistan management of green transportation and environmental pollution: a nonlinear ARDL analysis

**DOI:** 10.1007/s11356-021-12654-x

**Published:** 2021-02-06

**Authors:** Muhammad Tayyab Sohail, Sana Ullah, Muhammad Tariq Majeed, Ahmed Usman

**Affiliations:** 1 School of Public Administration, Xiangtan University, Xiangtan, Hunan China; 2 Quaid-i-Azam University, Islamabad, Pakistan; 3 Government College University, Faisalabad, Pakistan

**Keywords:** Air transportation, Railway transportation, CO_2_ emissions, NARDL, Pakistan

## Abstract

Modern advances in nonlinear modeling have exposed that nonlinear models yield more robust results compared with linear models. Research on the effect of air-railway transportation on environmental pollution has now arrived into a new way of asymmetry analysis and captured the real issue among the nexus. This study aims to inspect the asymmetric impact of air-railway transportation on environmental pollution in Pakistan by using annual time series data from 1991 to 2019. The findings show that positive shock in air passenger carried and railway passenger carried increases carbon emissions, which implies that 1% increase in air passenger carried (railway passenger carried) enhances environmental pollution by 0.21% (0.32%) in long run in Pakistan. While positive shock in railway passengers carried increases environmental pollution and negative shock in railway passengers carried decreases the environmental pollution in the short run. The outcomes have also confirmed the short- and long-run asymmetries in Wald statistics. The findings are country-specific and it would be regionally specific.

## Introduction

The transport sector plays a significant role in the development of an economy by connecting different sectors of the economy. Contrary to its positive role, it also comprises a major share of global carbon emissions. According to IEA (2018), transportation emissions comprise 25% of world carbon emissions, and that is 71% greater than that of emissions in 1990s. The transportation sector heavily relies on fossil fuels for energy input. The transport energy share in total energy was 23% in 1971 and rose to 29% in 2017. Moreover, oil has 93% share in total transport energy use (IEA 2019). Since transport sector excessively relies on fossil fuels and contributes to climate change and adversities of global warming, managing green transportation is one of the most pressing challenges of the contemporary world.

Among different transport modes, road transportation is referred as the major contributor to emissions. After road transportation, the aviation sector is the major contributor to emissions (Air Transport Action Group 2019). Emissions from aviation sector are, however, more environmentally harmful because they distort the environment at high altitudes and degrade environmental quality at surface. Half of the emissions are released near surface environment while remaining half of the emission are released above an altitude of 6000 m (Balkanski et al. 2010). If a return-trip flight from London to New York releases approximately 986-kg emission per passenger, then it surpasses the per capita emissions of 56 countries (Kommenda 2019). Thus, it can be inferred that aviation transportation pollutes the environment by emitting emission at the surface and at high altitude environment. Moreover, the transportation sector was one of the key sectors that require strategic management emphasized in 1997 Kyoto protocol.

The transport sector is considered the main source of economic growth and development. In the case of Pakistan, it contributes 10% of GDP and generates 6% of employment opportunities in the country. This sector also connects other sectors of the economy by promoting agglomerations, enhancing both national and cross-border trade, and enabling spatial transformation. The present transport and logistic system are not working efficiently and costing economy of Pakistan about 4–6% of GDP annually, which is a major constraint for the overall performance of the economy (Sánchez-Triana et al. 2013). Further, Pakistan’ exposure to climate-related adversities is ranked the highest in the world. The green transportation system is the need of present time as it is a key driver of environmental quality.

The transport sector of Pakistan faces numerous challenges that have diverse implications for energy and environmental problems. For example, the railway transport used to have the largest mean of transportation in the history of Pakistan. From 1955 to 1960, it has witnessed its peak by handling 73% of freight transportation, which fell to 4% in 2011. From 1991 to 2011, it has curtailed its track length by 11% that is 8875 to 7791 km. Total passengers carried also declined over the same period by 31% that is 84.9 to 58.9 million (GoP 2011). In 2019–2020, passengers carried declined to 39.4 million (GoP 2020). Another challenge with the transport sector of Pakistan economy is that vehicles run on fuels that have high sulfur content. Particularly, most fuels in Pakistan contain a sulfur level of 5000–10,000 parts per million. This level is substantially higher than that of Euro III and Euro IV emission standards. Some South Asian countries have adopted these Euro III or IV standards. In Pakistan, however, the adoption process is delayed for different reasons.

Transportation is ranked as the 2nd major source of energy consumption in Pakistan. The energy mix is heavily skewed towards conventional sources as 97% of energy comes from fossil fuels, almost 28% of which is consumed by transportation activities. Energy consumption-induced CO_2_ emissions in Pakistan increased from 68,242 (kt) in 1991 to 166,298 (kt) in 2014 (Khan and Majeed 2019). Currently, carbon emissions are growing by 6% each year and will become 400 Mt of CO_2_ equivalent (per year) by 2030 if things remain as it is (UNDP 2015). In Pakistan, public transport system is inefficient and exhibits low development, and as a consequence, private transportation is surging. Moreover, its middle class is growing, and therefore, the demand for private vehicles is consistently increasing (Rasool et al. 2019).

Following vision 2025, Pakistan has framed “National Transportation Policy (NTP)” which comprises many transportation sector development initiatives. The primary objective of this policy is to provide safe, secure, and affordable transport services to its citizens. Though NTP is aligned with environmentally friendly approaches, the government is facing practical applications of this policy. For instance, government of Pakistan has introduced rapid transportation system in three major cities of Pakistan, which is based on fossil fuels. Thus, public sector transportation will also escalate greenhouse gas (GHG) emissions in Pakistan like private vehicles. The major challenge to sustainable and green transport sector of Pakistan is its electricity crisis that needs to be settled on priority buses. Electric breakdown for longer hours has led to collapse of many industrial units in Pakistan. In such a situation, electric vehicles may not work successfully because electricity flow is not smooth.

The empirical studies based on diverse samples provide evidence on transport and emission nexus. Timilsina and Shrestha (2009) for Asian economies, Chandran and Tang (2013) for ASEAN countries, Zhang and Nian (2013) for China, Saboori et al. (2014) for OECD economies, Shahbaz et al. (2015) for Tunisia, Saidi and Hammami (2017) for a panel of 75 economies, Danish et al. (2018) and Shouket et al. (2019) for Pakistan, Du et al. (2019) for China, Ahmed et al. (2020) for India, Isik et al. (2020) for Turkey, Mangones et al. (2020) for Columbia, Hossain et al. (2020) for Bangladesh, and Go et al. (2020) for Malaysia explored transport and environment nexus. These studies assume a symmetric relationship between transportation and emissions. These studies provide diverse evidence on transport sector and the environment nexus.

The aforementioned mentioned studies have investigated the dynamic relationships between transport sector and emissions employing autoregressive distributive lag model (ARDL), dynamic ordinary least squares (DOLS), fully modified ordinary least squares (FMOLS), and generalized method of moment (GMM) methods of estimation. These studies model the dynamic relationships between transport and emissions by taking the controls of economic growth, population growth, urbanization, trade, and FDI, among others. These studies provided empirical evidence of the bases of assumed symmetric associations among the chosen variables, ignoring their hidden dynamic nonlinear associations. Since dynamic relationships among selected variables can be shaped by numerous elements such as social, economic, political, and global conditions, assuming symmetric associations can yield misleading inferences. Therefore, it is necessary to split positive and negative components of dynamic series to capture their diverse effects on environmental pollution. Moreover, in the presence of structural breaks, linear association may provide misleading results (Adedoyin et al. 2020a). In this scenario, we improve the extant literature by incorporating the positive and negative components of transport on environmental quality. In this way, we utilize the Shin et al. (2014) nonlinear ARDL approach and Hatemi-j (2012) asymmetric causality test to strengthen the existing literature on transport sector and carbon emission nexus.

To the best of authors’ knowledge, the earlier research studies have ignored the asymmetric effects of transport sector on environmental quality. Besides, few studies have explored the transport-environment nexus in the context of Pakistan. For example, Danish et al. (2018) and Shouket et al. (2019) have investigated transport environment nexus for Pakistan. However, these studies ignored the asymmetric associations between transport and emissions. Besides, these studies overlooked the role of air and rail transportation in shaping transport, energy, and environment nexus. Yet, to the authors’ knowledge, no past study has examined the effect of airline and railway industry on environmental pollution. Particularly, empirical research is missing in the framework of developing countries such as Pakistan. This study attempts to fill this research hiatus by utilizing asymmetric ARDL approach to estimate the nonlinear association between transport sector and environmental quality over the period 1980–2019.

Moreover, this study represents leading studies on Pakistan around the world that establishes asymmetries among the chosen time series indicators and a novel framework of analysis in environmental and transportation economics. The empirical findings of this study are useful for different stakeholders in the field of energy, environment, and transport such as passengers, academic researchers, energy economists, government officials, think tanks, and policy mangers. This research provides new fresh insights on transport sector and environment to environmentalists, government authorities, and policymakers to develop better strategies for environmental preservation. The findings of this research are helpful for other developing countries that are facing similar challenges in their transport sectors.

In the next sections, we have briefly reviewed the literature, provided a discussion on model, data, and methodology, results, and their discussion, and also offers suitable policy implications.

## Literature review

The transport sector has a substantial role in carbon emissions all over the world. The literature on transport and environment can be grouped into different categories such as country-specific studies, regional analysis, panel data analysis, among others. Some studies provide evidence on transport and environment nexus for a single country. In general, these studies show that transportation sector growth is costing environmental quality. However, these studies also indicate that the environmental effects of transport sector vary depending upon the modes and measures of transportation sector. Thus, these studies do not provide definite and clear relationships (Zhang and Nian 2013; Sohail et al. 2014, Shahbaz et al. 2015; Alshehry and Belloumi 2017; Kharbacha and Chfadi 2017; Du et al. 2019; Sohail et al. 2019; Ahmed et al. 2020).

Zhang and Nian (2013) investigated the evidence on transport sector-related emissions using regional data for China over the period 1995–2010. They employed Pedroni cointegration approach to test for the long-run associations between the variables in the panel data series. The results confirmed the positive impact of transport section on regional emissions in China. Their results exhibited that economic growth, population growth, and oil prices enhance emissions related to transport sector. Contrary to this, electricity consumption and freight turnover lower emissions. They showed similar findings for Eastern and Central regions of China.

Similarly, Du et al. (2019) empirically explored the transport-emission nexus for China during the years of 2002, 2007, and 2012, respectively. They showed positive impact of road length on emission while beneficial impact of rail length on emission mitigation. Du et al. (2019) further revealed that road, air, and rail infrastructure escalated carbon emissions in 2012. Furthermore, they provided evidence that transportation sector’s own demand does not contribute to emissions per se as the transport services demanded by other sectors of the economy contribute to emissions.

Alshehry and Belloumi (2017) explored transport-emission nexus in the framework of “Environmental Kuznets hypothesis (EKC)” hypothesis for Saudi Arabia. They employed ARDL-bound testing approach to explore the dynamic associations between emissions from the transport sector and economic growth. Their results, however, rejected the validity of EKC hypothesis for transport sector emissions. Similarly, Go et al. (2020) did not confirm the EKC for Malaysia over the period 1990–2017 while exploring transport-emission nexus. Contrary to this, Kharbacha and Chfadi (2017) supported EKC hypothesis for Morocco. They estimated the long-run relationships of transport sector, diesel consumption, the number of vehicles, and economic growth on CO_2_ emissions. Ahmed et al. (2020) investigated the transport CO_2_ emissions for India from 1980 to 2015. Their results show that economic growth and road sector energy consumption increase emissions. Besides, they show that road-related infrastructure boosts transport emissions while urbanization lowers missions. Some studies provide evidence on transport and environment nexus for regional groups of countries (Timilsina and Shrestha 2009; Chandran and Tang 2013; Liu et al. 2018). Timilsina and Shrestha (2009) explored CO_2_ emission drivers for Asian economies over the period 1980–2005. Their analysis suggested that economic growth, population growth, and energy consumption in transport sector significantly derive the emissions in Asian economies. Chandran and Tang (2013) explored the effects of economic growth, transport sector, and foreign direct investment on CO_2_ emissions using a panel of ASEAN-5 countries. The empirical results confirmed that positive impacts of transport sector and economic growth contribute on CO_2_ emissions whereas foreign direction investment has insignificant impact on emissions.

Some studies provide evidence on transport and environment nexus for a panel of countries (Saboori et al. 2014; Liddle 2015; Saidi and Hammami 2017; Andres and Padilla 2018; Erdogan et al. 2020). Saboori et al. (2014) investigated the long-run relationships of transport sector and carbon emissions for a panel of “Organization for Economic Cooperation and Development (OECD)” countries over the period 1960–2008. Their findings confirmed bidirectional causality between transport sector energy consumption and CO_2_ emissions. Liddle (2015) compiled the data for a panel of developed and developing economies EKC hypothesis between urban transport-related GHG emissions per capita income by controlling urban intensity and fuel prices into carbon emission function. The empirical estimate validated EKC hypothesis for CO_2_ emissions, nitrogen emissions, and volatile hydrocarbon whereas urban intensity and fuel prices lower these emissions.

In another panel data study, Saidi and Hammami (2017) findings suggest that freight transport, economic growth, energy use, and trade escalate environmental deterioration in the selected panel of countries. In a recent study, Andres and Padilla (2018) explored the drivers of GHG emissions for the transport sector employing panel data estimation approaches over the period 1980–2014. Their study covered a selected panel of European economies. The results confirmed a positive association between transport indicators and GHG emissions. They showed that both transport energy intensity and transport volume significantly contribute to GHG emissions. The aircraft flights are also responsible for global warming as they discharge hydrocarbons, carbon monoxide, and nitrogen oxide into the atmosphere. In this regard, Erdogan et al. (2020) examined the effects of air and rail transportation on environmental pollution exploiting the annual time series data for a panel of top 10 air passenger countries over the period 1995–2014 using robust panel estimators. Their estimates show that air transport contributes to emissions whereas railway transportation and urban growth mitigate the emissions over the study period. They emphasize the need for clean air sector operations. Saleem et al. (2018) investigate air-railway transportation and environmental pollution nexus for Next-11 countries from 1975 to 2015. They found the emissions escalating effect of air transport passengers carried while air transport has a negative relationship with the natural resource rents.

There are few studies that provide evidence on transport and environment nexus in the context of Pakistan (Danish et al. 2018; Shouket et al. 2019). Danish et al. (2018) investigated the dynamic long-run relationships between economic growth, transport-related energy consumption, and environmental quality of Pakistan. They measured environmental quality by sulfur dioxide (SO2). Their results confirmed a positive association between energy use in the transport sector and environmental quality. Shouket et al. (2019) analyze the environmental effects of air and railway transportation in the context of Pakistan over the period 1975–2016. Their findings suggest that railway passengers carried has a positive impact on emissions while air-railway transportation and travel services deteriorate environmental quality by depleting natural resources.

In nutshell, the aforementioned discussion suggests that transportation sector plays a significant role in influencing environmental quality. The extant literature on transport and environmental nexus is not conclusive. Collectively, these studies indicate that the environmental pollution effects of transport sector are sensitive to the modes and measures of transportation sector. Thus, these studies do not provide definite and clear relationships. The available literature has excessively focused on road transport while the role of rail and air on emissions is not explored for Pakistan. Furthermore, these studies have provided evidence assuming a symmetric relationship between transport sector and the environment.

## Model, methodology, and data

Most of the empirical studies in the previous section have identified green transportation to be the main determinant of air quality. Therefore, we follow the theoretical and empirical literature and rely upon the following carbon emission model:

1 $$ {\mathrm{CO}}_{2,\mathrm{t}}={\varphi}_0+{\varphi}_1{\mathrm{APC}}_{\mathrm{t}}+{\varphi}_2{\mathrm{RPC}}_{\mathrm{t}}+{\varphi}_3{\mathrm{GDP}}_{\mathrm{t}}+{\varphi}_4{\mathrm{POP}}_{\mathrm{t}}+{\varepsilon}_{\mathrm{t}} $$

where subscripts t indicate years, *φ*_0_, *φ*_1_, *φ*_2_, *φ*_3_, and *φ*_4_ are the parameters for estimation; CO_2, t_ denotes the carbon emissions, APC_t_ denotes the air passenger carried, RPC_t_ denotes the railway passenger carried, and GDP_t_ denotes the gross domestic product, POP_t_ denotes the population growth, and *ε*_t_ is the random term, respectively. All data obtained from World Bank. Similarly, equation (1) is supposed to depend on air passenger carried, railway passenger carried, GDP, and population growth. Since increased APC and RPC lead to more green economic activities, we expect estimates of *φ*_1_ and *φ*_2_ to be negative in environmental pollution, while equation (1) gives the long-run estimates by using any method. Pesaran et al. (2001) announce a method that offers short-term and long-term estimates in one step. We follow their econometric approach to achieve the following error-correction models:

2 $$ {\Delta \mathrm{CO}}_{2,\mathrm{t}}={\omega}_0+\sum \limits_{\mathrm{k}=1}^{\mathrm{n}}{\beta}_{1\mathrm{k}}{\Delta \mathrm{CO}}_{2,\mathrm{t}-\mathrm{k}}+\sum \limits_{\mathrm{k}=0}^{\mathrm{n}}{\beta}_{1\mathrm{k}}{\Delta \mathrm{APC}}_{\mathrm{t}-\mathrm{k}}+\sum \limits_{\mathrm{k}=0}^{\mathrm{n}}{\beta}_{1\mathrm{k}}{\Delta \mathrm{RPC}}_{\mathrm{t}-\mathrm{k}}+\sum \limits_{\mathrm{k}=0}^{\mathrm{n}}{\beta}_{1\mathrm{k}}{\mathrm{GDP}}_{\mathrm{t}-\mathrm{k}}+\sum \limits_{\mathrm{k}=0}^{\mathrm{n}}{\beta}_{1\mathrm{k}}{\mathrm{POP}}_{\mathrm{t}-\mathrm{k}}+{\omega}_1{\mathrm{CO}}_{2,\mathrm{t}-1}+{\omega}_2{\mathrm{APC}}_{\mathrm{t}-1}+{\omega}_3{\mathrm{RPC}}_{\mathrm{t}-1}+{\omega}_4{\mathrm{GDP}}_{\mathrm{t}-1}+{\omega}_5{\mathrm{POP}}_{\mathrm{t}-1}+{\varepsilon}_{\mathrm{t}} $$

In the above model, the coefficients assigned to “Δ” variables indicate short-run impacts, and those assigned to “lagged level” variables indicate long-run impacts. However, to compare the long-run impacts in equation (2), estimates of *ω*_2_–*ω*_5_ must be normalized on *ω*_1_ in equation (2). To establish cointegration, Pesaran et al. (2001) suggest employing the *F* test to verify the joint level of significance of lagged variables, which indicates a sign of cointegration. In this context, the *F* test has tabulated new critical values in our analysis. Equation (2) is error-correction econometric models in which green transportation is supposed to respond to changes in environmental pollution in symmetric manner. Freshly, Shin et al. (2014) have modified such models and supposed to asymmetric response of exogenous variables on the outcome variable. Since our basic aim is to evaluate the asymmetric impacts of green transportation, we follow Shin et al. (2014) econometric approach by creating the two new time series variables as follows:

3 $$ {{\mathrm{APC}}^{+}}_{\mathrm{t}}=\sum \limits_{\mathrm{n}=1}^{\mathrm{t}}\Delta  {{\mathrm{APC}}^{+}}_{\mathrm{t}}=\sum \limits_{\mathrm{n}=1}^{\mathrm{t}}\max\ \left(\Delta  {{\mathrm{APC}}^{+}}_{\mathrm{t}},0\right) $$

4 $$ {{\mathrm{APC}}^{-}}_{\mathrm{t}}=\sum \limits_{\mathrm{n}=1}^{\mathrm{t}}\Delta  {{\mathrm{APC}}^{-}}_{\mathrm{t}}=\sum \limits_{\mathrm{n}=1}^{\mathrm{t}}\min\ \left(\Delta  {{\mathrm{APC}}^{-}}_{\mathrm{t}},0\right) $$

5 $$ {{\mathrm{RPC}}^{+}}_{\mathrm{t}}=\sum \limits_{\mathrm{n}=1}^{\mathrm{t}}\Delta  {{\mathrm{RPC}}^{+}}_{\mathrm{t}}=\sum \limits_{\mathrm{n}=1}^{\mathrm{t}}\max\ \left(\Delta  {{\mathrm{RPC}}^{+}}_{\mathrm{t}},0\right) $$

6 $$ {{\mathrm{RPC}}^{-}}_{\mathrm{t}}=\sum \limits_{\mathrm{n}=1}^{\mathrm{t}}\Delta  {{\mathrm{RPC}}^{-}}_{\mathrm{t}}=\sum \limits_{\mathrm{n}=1}^{\mathrm{t}}\min\ \left(\Delta  {{\mathrm{RPC}}^{-}}_{\mathrm{t}},0\right) $$

where APC^+^_t_ (RPC^+^_t_) is the new time-series variable of positive changes in air passenger carried (railway passenger carried) which infers only increased green transportation. Similarly, APC^−^_t_ (RPC^−^_t_) is the new time-series variable of negative changes in air passenger carried (railway passenger carried) which infers only deceased green transportation. In the next step, we move back to equation (2) to replace positive and negative changes in APC and RPC. The extended error-correction models are

7 $$ {\Delta \mathrm{CO}}_{2,\mathrm{t}}={\upalpha}_0+\sum \limits_{\mathrm{k}=1}^{\mathrm{n}}{\beta}_{1\mathrm{k}}{\Delta \mathrm{CO}}_{2,\mathrm{t}-\mathrm{K}}+\sum \limits_{\mathrm{k}=0}^{\mathrm{n}}{\uppi}_{1\mathrm{k}}\Delta  {{\mathrm{APC}}^{+}}_{\mathrm{t}-\mathrm{k}}+\sum \limits_{\mathrm{k}=0}^{\mathrm{n}}{\delta}_{1\mathrm{k}}\Delta  {{\mathrm{APC}}^{-}}_{\mathrm{t}-\mathrm{k}}+\sum \limits_{\mathrm{k}=0}^{\mathrm{n}}{\eta}_{1\mathrm{k}}\Delta  {{\mathrm{RPC}}^{+}}_{\mathrm{t}-\mathrm{k}}+\sum \limits_{\mathrm{k}=0}^{\mathrm{n}}{\phi}_{1\mathrm{k}}\Delta  {{\mathrm{RPC}}^{-}}_{\mathrm{t}-\mathrm{k}}+\sum \limits_{\mathrm{k}=0}^{\mathrm{n}}{\mu}_{1\mathrm{k}}{\mathrm{GDP}}_{\mathrm{t}-\mathrm{k}}+\sum \limits_{\mathrm{k}=0}^{\mathrm{n}}{\lambda}_{1\mathrm{k}}{\mathrm{POP}}_{\mathrm{t}-\mathrm{k}}+{\omega}_1{\mathrm{CO}}_{2,\mathrm{t}-1}+{\omega}_2{{\mathrm{APC}}^{+}}_{\mathrm{t}-1}+{\omega}_3{{\mathrm{APC}}^{-}}_{\mathrm{t}-1}+{\omega}_4{{\mathrm{RPC}}^{+}}_{\mathrm{t}-1}+{\omega}_5{{\mathrm{RPC}}^{-}}_{\mathrm{t}-1}+{\omega}_6{\mathrm{GDP}}_{\mathrm{t}-1}+{\omega}_7{\mathrm{POP}}_{\mathrm{t}-1}+{\varepsilon}_{\mathrm{t}} $$

Such types of models are commonly known as nonlinear ARDL models, Shin et al. (2014) establish that both the linear and nonlinear ARDL models could be estimated by OLS methods with similar diagnostic tests. For some other econometric application of nonlinear ARDL models, see Ullah et al. (2020a), Ullah et al. (2020b), and Ullah et al. (2020c). After the estimation (7), we can perform some extra test of asymmetric assumptions. First, positive shocks APC^+^_t_ (RPC^+^_t_) is different lag order than the negative shock APC^−^_t_ (RPC^−^_t_), implying that short-run asymmetry will be established. Second, in other words, if *π*_1k_ ≠ *δ*_1k_ and *η*_1k_≠ *ϕ*_1k_in equation (7), “adjustment asymmetry” will be supported. Similarly, in third, we confirm the short- and long-run cumulative or impact asymmetries by employing the Wald test.

## Results and discussion

Before starting a formal discussion on the estimates of our variables, we need to decide a few things. First of all, our variables should be stationary at a level or a first difference. Though the unit root testing is not a mandatory condition for applying ARDL methodology but, for our satisfaction, we have confirmed with the help of augmented Dickey Fuller (ADF) and Phillips-Perron (PP) unit root tests that none of the variables included is I(2). The descriptive statistics and results of both the unit root tests are reported in Table 1. Secondly, and as our data is annual and observations are 27, we have applied a maximum of two lags. Lastly, to select the appropriate lag length, out of a maximum two, we have relied upon the Akaike information criteria (AIC).

**Table 1:** Unit root tests

	Descriptive statistics		Unit root tests			
			ADF		PP	
	Mean	Std. Dev	I(0)	I(1)	I(0)	I(1)
CO_2_	0.808	0.116	−0.75	−6.34**	−0.63	−6.33**
GDP	898.6	112.5	−0.17	−3.11**	−0.12	−3.11**
POP	2.470	0.305	−0.17	−8.46**	−0.83	−4.23**
APC	6067764	130891	−0.51	−5.96**	−0.51	−5.96**
RPC	19768	3856	−3.25**		−3.51**	

A test of nonlinearity is used through which the analysis can confirm whether the modeling approach should be linear or nonlinear. The test, developed by Brock et al. (1996), has been frequently used by studies, such as Kim et al. (2003), Sheikh et al. (2020), and Galadima and Aminu (2020). According to null hypothesis of the test, the series is linearly dependent against the alternative hypothesis which states that series is not linearly dependent. If the null hypothesis is rejected, then the linear model is mis-specified and we can apply the nonlinear methodology. The results, reported in Table 2, reject the null hypothesis and confirm the nonlinear nature of both the series, i.e., APC and RPC.

**Table 2:** BDS test of nonlinearity

Dimension	BDS statistic	Std. error	z-statistic	Prob.
APC				
2	0.073**	0.014	5.079	0.000
3	0.093**	0.023	3.955	0.000
4	0.109**	0.029	3.744	0.000
5	0.070**	0.031	2.249	0.024
6	-0.088**	0.031	-2.816	0.005
RPC				
2	0.199**	0.018	10.93	0.000
3	0.334**	0.029	11.21	0.000
4	0.419**	0.036	11.48	0.000
5	0.466**	0.039	11.89	0.000
6	0.479**	0.038	12.33	0.000

First, we discuss the results of the linear models reported in Table 3. From panel A, we see that short-run estimate attached to GDP is significant in both models. From this finding, we confer that as the people of Pakistan become richer, they contribute more to polluting the environment. The likely reason could be that economic growth in Pakistan is largely based on non-renewable energy sources whereas Rafindadi and Ozturk (2017) argued that renewable energy sources are essential for green growth. The short-run effects of population growth on CO_2_ emissions are insignificant in both models which imply that population growth does not contribute to CO_2_ emissions in short run. Likewise, in short run, the estimated coefficients of air and railway transport variables are insignificant which suggest that these variables do not have any noticeable impact in polluting the environment in a shorter period.

**Table 3:** ARDL and NARDL estimates

	ARDL-APC		NARDL-APC		ARDL-RPC		NARDL-RPC	
	Coefficients	t-stats	Coefficients	t-stats	Coefficients	t-stats	Coefficients	t-stats
Panel A: short-run estimates								
ΔAPC_t_	−0.05	1.14						
ΔAPC_*t*_^+^			0.06	0.92				
ΔAPC_*t* − 1_^+^			0.14**	2.01				
ΔAPC_*t*_^−^			−0.05	0.83				
ΔRPC_t_					−0.07	1.08		
ΔRPC_*t*_^+^							0.41**	4.41
ΔRPC_*t*_^−^							−0.66**	3.78
ΔGDP_t_	0.52**	1.93	1.04**	4.98	0.46**	2.36	0.63**	3.32
ΔPOP_t_	0.09	0.31	0.08	0.27	−0.98	0.59	0.05	0.26
Panel B: long-run estimates								
APC	0.16**	2.13						
APC^+^			0.21*	1.86				
APC^−^			−0.11	1.00				
RPC					0.19**	8.18		
RPC^+^							0.32**	7.29
RPC^−^							0.08	1.27
GDP	1.38**	4.27	1.49**	4.89	0.82**	9.89	0.65**	5.82
POP	0.04	0.14	0.07	0.01	0.13	1.62	0.03	0.29
C	−7.01**	3.49	−0.73	0.83	−2.75**	9.35	−1.05	7.63
Panel C: diagnostic statistics								
ECMt-1	−0.68**	4.02	−1.96**	8.57	−1.20**	8.23	−1.34**	6.81
ADJ-R2	0.97		0.97		0.98		0.98	
*F* test	5.77		5.60**		11.9**		16.4**	
LM	1.49		1.03		2.51		2.11	
RESET	1.57		0.56		0.06		0.01	
CUSUM	S		S		S		S	
CUSUMSQ	S		S		S		S	
Wald-SR-APC			9.981**					
Wald-LR-APC			18.00**					
Wald-SR-RPC							12.15**	
Wald-LR-RPC							3.871**	

* and ** denote 10% and 5% levels of significance, respectively. The critical values of RESET, LM, and Wald tests at the 10% level of significance are 2.70 and at 5% level 3.84

Panel B of Table 3 reports long-run estimates of the linear model. In both the models, increased GDP per capita in Pakistan exerted a positive impact on the CO_2_ emissions in the country. From the estimates, we see that a 1% increase in GDP per capita increases the CO_2_ emissions by 1.32% in air transport model and 0.82% in railway transport model. According to Zaman and Abd-el Moemen (2017), economic growth is the main contributor to degrade environmental quality. Some recent studies also suggest that economic growth is the key determinant of emissions (Dogan and Ozturk 2017; Baloch et al. 2020; Majeed and Tauqir 2020; Adedoyin et al. 2020b). Hence, we can say that economic development in Pakistan is not sustainable and coming at the cost of environmental degradation. However, population growth does not contribute significantly to increasing or decreasing CO_2_ emissions. On the other side, a 1% increase in air transport passengers carried to increase the CO_2_ emissions by 0.16%, whereas a 1% increase in railway passengers increases the CO_2_ emissions by 0.19%. Hence, we confer that increased transportation through airplanes and rails deteriorates the environmental quality as enhanced transportation requires more energy consumption and generates few other activities that add carbon footprints in the environment. These findings are also supported by a few other studies like Shouket et al. (2019) and Alshehry and Belloumi (2017).

The validity of the long-run results depends upon whether the results are co-integrated or not. To that end, we trust the tests of co-integration, i.e., bounds *F* test or error correction modeling (ECM_t-1_) reported in panel C. The *F* test confirms the co-integration in the railway transport model; however, in the air transport model, we see the negative and significant estimate attached to ECM_t-1_ which confirms the co-integration in the model as well. Few other diagnostic checks are reported in panel C, which includes a test of first-order serial correlation, i.e., Langrage multiplier (LM) test, a test of misspecification, i.e., Ramsey’s RESET test, a test of parametric stability, i.e., CUSUM and CUSUMSQ. The stability of the parameters is represented by S and instability is represented by US. All these tests confirm that our models are free from serial correlation, correctly specified and stable.

The foremost objective of our study is to see the asymmetric impact of air and railway transportation on environmental quality in Pakistan. The asymmetric estimates of both models are reported in Table 3. From Panel A, we gather that the short-run estimates attached to the GDP in air transport model and railway transport model are positive and significant. Conversely, the estimates attached to POP are insignificant in both the models. After that, we turn our attention to short-run nonlinear estimates of our transportation variables. The estimate connected to a positive component of the variable of air transport passenger carried is positively significant at a previous lag and insignificant at a current lag. Contrariwise, the negative part of air transport passengers carried does not have a significant impact on the CO_2_ emissions in Pakistan. This is an indication of short-run impact asymmetric effects of air transportation on the CO_2_ which is confirmed by the significant WALD-SR reported in panel C of Table 3. Similarly, in railway transport model, the asymmetric estimates attached to RPC variables have opposite signs which are a confirmation of short-run asymmetric impacts and also supported by the significant statistics of the WALD-SR.

**Fig. 1:**
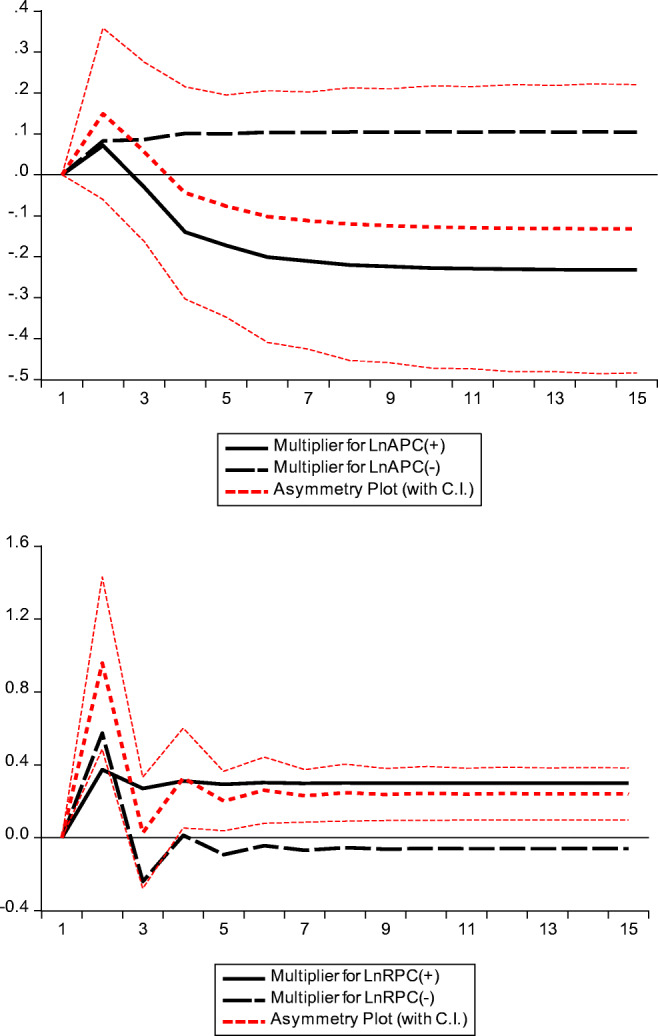
Dynamic multiplier graphs for green transportation

In long run, the estimate attached to APC^+^ is positive; however, the estimate attached to APC^−^ is insignificant. This result implies that in case of increased passenger carried by airplanes the CO_2_ level in Pakistan’s environment sees an upsurge and in the case of decreased passenger traveled through air traffic, the CO_2_ emissions did not increase or decrease significantly. Moreover, this result confirms the asymmetric impacts of increased and decreased passengers carried by airplanes on the environmental quality of Pakistan which is also represented through significant statistics of WALD-LR. Similarly, the nonlinear estimates of RPC variable have shown a different impact on the environmental quality in Pakistan, i.e., RPC^+^ has a positive and significant impact and RPC^−^ has an insignificant impact which confirms the asymmetry in the effects of increased and decreased components of the variables. Besides, the estimates of few other long-run variables are also reported in the panel B of Table 3, which have the same explanation as already discussed in our linear models, hence, no need to further elaborate them. Once again, the legitimacy of long-run results is to be confirmed through one or the other co-integration tests, and from seeing the values of F-statistics, we confirm that both the nonlinear models are also co-integrated. Similarly, just like our linear models, few other diagnostic statistics are reported in panel C which confirms that our nonlinear models are not suffering from autocorrelation, correctly specified and parametrically stable. The dynamic multiplier graphs for green transportation are reported in Fig. 1.

## Conclusion and policy implications

Public transportation has been enlarged in Pakistan in the last two decades. Public transportation is said to affect environmental pollution in either direction. Theoretical developments and econometric results of earlier studies do support these views. However, newly empirical research has followed a novel direction of examining the possibility of an asymmetric response of environmental pollution to public transportation. This literature on public transportation and environmental pollution is incomplete and infancy in economics. In this light, the aim of the study is to test the dynamic asymmetric linkages between air-railway transportation and environmental pollution in the context of Pakistan by employing the modern NARDL approach from the sample period 1990 to 2019. The symmetric effects are estimates through linear ARDL methodology of Pesaran et al. (2001) and assess the asymmetric effects by using the nonlinear ARDL methodology of Shin et al. (2014). The symmetric findings show that air passenger carried and railway passenger carried have a positive significant effect in long run, but this effect is insignificant in the short run in the case of Pakistan. The asymmetric findings show that positive shock in air passenger carried and railway passenger carried increases carbon emissions, which implies that 1% increase in air passenger carried (railway passenger carried) enhances the environmental pollution by 0.21% (0.32%) in long run in Pakistan. While positive shock in railway passengers carried increases the environmental pollution and negative shock in railway passengers carried decreases the environmental pollution in short run. The railway passenger carried effects are mostly large compared with air passenger carried in short and long in the symmetric and asymmetric analysis. The results also show that GDP is associated with carbon emissions in short and long run, while the population has an insignificant effect on environmental pollution in short and long run in symmetric and asymmetric models.

Based on these findings, we suggest some policy implications for the Pakistan’s economy. The green transportation system should be introduced to alleviate environmental pollution. Logistics activities should be environmental-friendly and provide awareness to the public and private sector about green logistics and the environment in Pakistan. Advance pollution-free diesel, petrol, and CNG vehicle engine introduce in small and big cities in the transportation sector. Green and efficient fuel substitution strategy must be embraced by Pakistan in a high inflation period in the economy. The Pakistani authorities should implement green packaging, green transportation, and green supply chain design in the economy to promote their green economic growth. The study also decided that government authorities should take serious steps to re-define the physical infrastructure of the transport sector in order to encourage environmental quality agenda by announcing green and digital transportation systems, which is authoritative for the digital economy’s long-term sustainable environmental development. These results are country-specific and generalize the economic policy implications for other developing economies for environmental sustainability.
